# Developing an online positive psychology application for people with bipolar disorder; ‘How expectations of consumers and professionals turned into an intervention.’

**DOI:** 10.1192/j.eurpsy.2022.1039

**Published:** 2022-09-01

**Authors:** B. Geerling, S. Kelders, A. Stevens, R. Kupka, E. Bohlmeijer

**Affiliations:** 1 Dimence, Scbs, Deventer, Netherlands; 2 University Twente, Bms, Enschede, Netherlands; 3 Amsterdam UMC, VU medical centre, Psychiatry, Amsterdam, Netherlands

**Keywords:** co-creation, positive psychology, intervention development, bipolar disorder

## Abstract

**Introduction:**

In Bipolar Disorder (BD), people report a lower quality of life and lower levels of well-being than the general population. Additionally, patients with bipolar disorder have unmet needs which are closely linked to elements of positive psychology.

**Objectives:**

The current study aimed to gain insight from patients with BD and care professionals about their thoughts of online Positive Psychology Interventions (PPI) to develop an app containing PPI’s for people with BD.

**Methods:**

The study is conducted in accordance with the CeHRes roadmap principles. Data were collected by focus groups, questionnaires, rapid prototyping and online feedback from the participants. Three focus groups meetings (FGM) were held with consumers (8) and professionals (5).

**Results:**

The FGM reveals a need for positive psychology interventions to cover some of the unmet needs that can be applied in an app in addition to the guidelines-advised treatment. Patients and professionals expect that PPIs in the current treatment in BD can meet some of the needs that are currently still unmet, specifically offering hope, increasing self-esteem, expressing feelings, acceptation and preventing social isolation. The process of contextual inquiry and value specification is helpful to guide this process.

**Conclusions:**

The consensus on the different topics about the use of positive psychology intervention shows that both consumers and professionals underline the importance of applying PPI’s in BD. The use during subsyndrome and mild depressive episodes seem the most beneficial periods for patients with BD. A more extended study has to be conducted to confirm if these findings are more generalizable

**Disclosure:**

No significant relationships.

